# Hypovolemic Shock Caused by Massive Renal Hematoma After a Third Consecutive Extracorporeal Shockwave Lithotripsy Session: A Case Report

**DOI:** 10.1089/cren.2016.0127

**Published:** 2016-12-01

**Authors:** Loic Sermeus, Kathy Vander Eeckt, Dieter Ost, Marcel Van Den Branden

**Affiliations:** ^1^ Faculty of Medicine, Katholieke Universiteit Leuven, Leuven, Belgium.; ^2^ AZ Sint Blasius, Dendermonde, Belgium.

**Keywords:** extracorporeal shockwave lithotripsy, hypovolemic shock, interval, multiple sessions, renal hematoma, complication

## Abstract

Extracorporeal shockwave lithotripsy (SWL) is a commonly used technique for treating urinary calculi. Although noninvasive, highly effective, and widely accepted, SWL is not without complications. Next to fragmenting the calculi, the surrounding tissue is damaged, which can result in renal hematoma, a well-described complication. In most cases, the collateral tissue damage is mild and resolves with conservative treatment. However, rarely, severe complications may arise. Here we present a case of a 46-year-old male who developed a massive hematoma, both subcapsular and retroperitoneal, after a third consecutive SWL session, resulting in hypovolemic shock. Different probable causes are proposed, of which one cause, the length of the interval between SWL sessions, is not yet studied properly. Probably, short intervals keep the damaged tissue from healing sufficiently, as proposed in our case. Possibly, life-threatening situations can be avoided if more evidence-based guidelines are available.

## Case Presentation

### Clinical history

A 46-year-old male presented to the emergency department with colicky pain in the left dorsal lumbar region. There was no radiating pain or other associated symptoms, except for the urge to move.

In his medical history, our patient had a vasectomy and an earlier episode of calculi of the urinary tract. Conservative treatment of the latter had led to spontaneous evacuation of the calculi. Allopurinol 300 mg once daily was started for secondary prevention and was faithfully taken until now. At present, the patient also took sertraline 100 mg once daily. No anticoagulant medication was taken.

### Physical examination

On clinical examination, left kidney percussion pain could be elicited. No other thoracic or abdominal abnormalities were obtained during inspection, auscultation, percussion, or palpation. Systemic parameters were normal, except for the blood pressure, which was elevated (200/130 mm Hg). Body temperature was not elevated.

### Diagnosis

Owing to suspicion of a renal colic, imaging was performed. X-ray of the abdomen revealed two dense opacities on the left side, later specified as urinary calculi with ultrasound imaging. One was located in the left kidney interpolar region. The other had a diameter of 12.7 mm and was obstructing the left ureteropelvic junction (UPJ), causing hydronephrosis.

### Intervention

In the acute phase, nonsteroidal anti-inflammatory drugs (ketorolac 30 mg) were administered intravenously and the patient was admitted to the urologic department. The asymptomatic left kidney interpolar calculus was left untreated and is not further discussed in this report. The symptomatic calculus in the left UPJ was fragmented with two consecutive sessions of extracorporeal shockwave lithotripsy (SWL) under general anesthesia. Good localization of the calculus was achieved with X-ray imaging. In each session, 3000 pulses with a frequency of 90/min and a maximal voltage of 30 kV were administered using a Siemens Modularis Variostar. Routine perioperative imaging showed effective disintegration of the calculus. No postoperative complications occurred and the patient left the hospital in good general condition.

### Follow-up

The patient was seen 7 days after the treatment at the outpatient clinic. He still reported much discomfort, a need for painkillers, and hematuria. Radiographic evaluation showed a disintegrated lithiasis in the left UPJ of ∼5 mm diameter and distal Steinstrasse in the same ureter. Further evacuation of these fragments was expected.

At day 14, our patient was relieved of his complaints after spontaneous evacuation of the fragments. On radiographic image, the Steinstrasse had disappeared, although the small calculus in the UPJ was still present. No spontaneous evacuation was expected and because of the high risk for recurrent symptomatic obstruction, a third SWL session was planned. The same treatment protocol was used with a good localization of the stone.

### Outcome

**FIG. 1.**
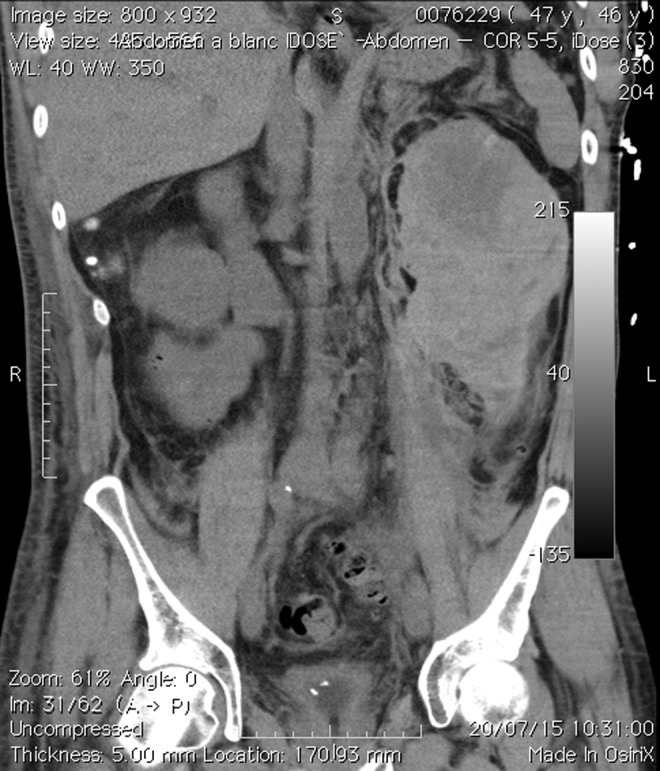
Coronal CT image of a massive subcapsular and retroperitoneal renal hematoma in the left hemiabdomen after a third consecutive SWL treatment of a calculus in the left UPJ. SWL, extracorporeal shockwave lithotripsy; UPJ, ureteropelvic junction.

**FIG. 2.**
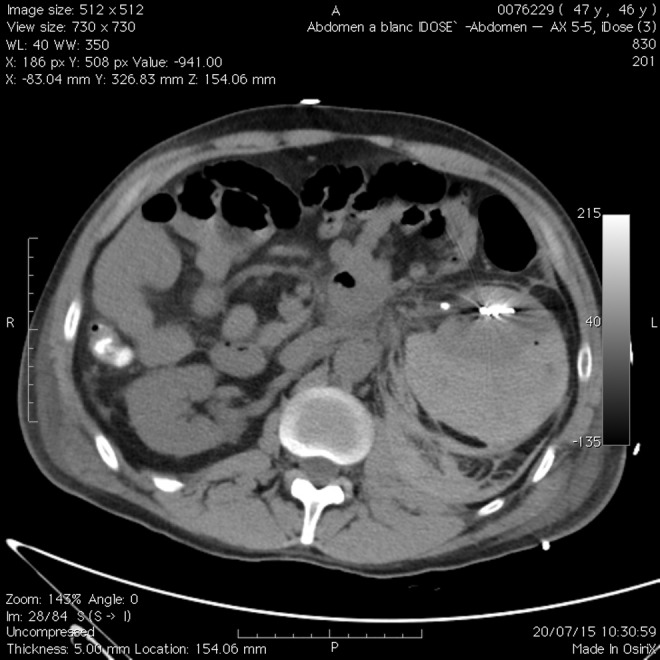
Axial CT image of a resorbing massive subcapsular and retroperitoneal renal hematoma in the left hemiabdomen, caused by a third consecutive SWL treatment of a calculus in the left UPJ, after coiling of the bleeding site.

During recovery, the patient developed acute and severe pain on inspiration in the left lumbar region. Immediately, our patient went into shock. An urgent computed tomography with contrast showed a massive hematoma with both a subcapsular and a retroperitoneal component surrounding the left kidney (Fig. 1). Contrast emerging at the left kidney interpolar region suggested an active bleeding near the renal artery. Effective embolization of this bleeding site with a pushable coil (Fig. 2) and transfusion was life saving.

## Discussion

SWL is considered “the least invasive treatment modality with high success rates for urinary calculi.”^1^ However, still many complications occur because of this procedure. The most common are colicky pain (40%), macroscopic hematuria (32%), and Steinstrasse (24.2%), of which the majority are considered mild.^1^ These complications were present in our patient after the first two SWL sessions.

Hematomas are less common with a reported incidence of 4.6% to 25% and symptomatic hematomas only occur in <1%.^1,2^ In rare cases, patients with this latter complication present with life-threatening hemodynamic instability.^1,3^ Hematomas after SWL are attributed to the tissue damaging effect of the administered pulses. Several factors may contribute. First, there are process-specific factors, for example, the properties and settings of the lithotripter, the process of cavitation inherent to SWL, or respiratory movements of the patient.^2,3^ Other factors are patient-specific, for example, bleeding diathesis, antiplatelet activity, diabetes mellitus, and hypertension.^1^ In the present case, elevated blood pressure might be considered a contributing factor caused by elevated mechanical stress on the blood vessel walls. It is postulated that if blood pressure exceeds 160/100 mm Hg, rescheduling should be considered till after effective antihypertensive therapy.^3^ However, to save the kidney, at risk because of hydronephrosis, an acute intervention was required.

At first glance, it seems that the wrong calculus was focused during the third SWL session, according to the localization of the bleeding site. However, postprocedure imaging showed disintegration of the calculus located in the left UPJ, which confirms an effective procedure. The stone in the left kidney interpolar region was still present.

Except for hypertension, no other known contributing factors could explain the occurrence of hematoma in this patient. However, tissue damage during SWL could be correlated to the number of effective SWL sessions in a short not yet specified period of time. Although the standard protocol of the medical institution was followed, three successive SWL sessions were performed in 16 days, with the first and second interval being 2 and 14 days, respectively. This could be explained by cumulative damage with insufficient time for the tissue to heal this. One prospective study with 857 patients reported a case in which the possible correlation between the length of the interval between successive SWL sessions and the occurrence of an asymptomatic renal hematoma was mentioned. Although no statistically significant evidence could be obtained, the authors “discourage the performance of more than two ESWL sessions ipsilaterally within 7 days. Two treatments within 3 days appear to be safe.”^3^ The European Association of Urology provides a low evidence guideline (grade 4): “There are no conclusive data on the intervals required between repeated ESWL sessions. However, clinical experience indicates that repeat sessions are feasible (within 1 day for ureteral stones).”^4^

## Conclusion

Symptomatic renal hematoma is a rare complication of SWL for urinary calculi. The possibility of successive SWL sessions with a short interval being a risk factor for its occurrence has been proposed. Until now, no qualitative evidence or guidelines have been published. As life-threatening situations are seen occasionally, further research is required.

## Abbreviations Used

CT: computed tomography
SWL: extracorporeal shockwave lithotripsy
UPJ: ureteropelvic junction

## References

[1] Salem S, Mehrsai A, Zartab H, Shahdadi N, Pourmand G Complications and outcomes following extracorporeal shock wave lithotripsy: A prospective study of 3,241 patients. Urol Res 2010;38:135–142 20016885 10.1007/s00240-009-0247-8

[2] Skolarikos A, Alivizatos G, de la Rosette J Extracorporeal shock wave lithotripsy 25 years later: Complications and their prevention. Eur Urol 2006;50:981–990 16481097 10.1016/j.eururo.2006.01.045

[3] Schnabel MJ, Gierth M, Chaussy CG, Dötzer K, Burger M, Fritsche HM Incidence and risk factors of renal hematoma: A prospective study of 1,300 SWL treatments. Urolithiasis 2014;42:247–253 24419328 10.1007/s00240-014-0637-4

[4] Türk C, Knoll T, Petrik A, et al. Pocket Guidelines on urolithiasis. Eur Urol 2014;40:362–371

